# Causal relationship of genetically predicted gut microbiota with thyroid cancer: a bidirectional two-sample mendelian randomization study

**DOI:** 10.3389/fendo.2024.1284472

**Published:** 2024-03-01

**Authors:** Xiaohe Sun, Shuai Chen, Shuoqi Zhao, Jingwen Wang, Haibo Cheng

**Affiliations:** ^1^ Jiangsu Province Hospital of Chinese Medicine, Affiliated Hospital of Nanjing University of Chinese Medicine, Nanjing, Jiangsu, China; ^2^ Jiangsu Collaborative Innovation Center of TCM Prevention and Treatment of Tumor, The First Clinical Medical College, Nanjing University of Chinese Medicine, Nanjing, Jiangsu, China; ^3^ The Second Affiliated Hospital of Nanjing University of Chinese Medicine, Nanjing, Jiangsu, China

**Keywords:** thyroid cancer, gut microbiota, mendelian randomization, causality, genetics

## Abstract

**Background:**

Previous investigations have demonstrated a correlation between the composition of gut microbiota and the development of thyroid cancer (TC). Nonetheless, there was no consensus on the causal effect of gut microbiota composition on TC risk. Therefore, the present study aimed to perform a bidirectional two-sample Mendelian randomization (MR) analysis to explore potential causal associations between gut microbiota and TC risk.

**Methods:**

Utilizing data from the MiBioGen consortium’s genome-wide association studies (GWAS) meta-analysis involving a sample size of 18,340, we identified instrumental variables for 211 gut microbiota taxa. The summary statistics for TC was from relevant large-scale GWAS conducted by the FinnGen consortium. In the first stage, the Inverse-variance weighted (IVW) method was used as the primary estimate method, and the stability of estimations was tested by a battery of sensitivity analyses. In the second stage, a reverse MR analysis was applied to determine whether reverse causality existed.

**Results:**

According to the IVW method, we identified 9 genetically predicted gut microbiota that were causally correlated with TC risk. Among them, we observed a positive causal effect of *Family Christensenellaceae* (OR = 1.664, 95% CI: 1.103–2.511, *P* = 0.015), *Family Victivallaceae* (OR = 1.268, 95% CI: 1.009–1.594, *P* = 0.042), *Genus Methanobrevibacter* (OR = 1.505, 95% CI: 1.049–2.159, *P* = 0.027), *Genus Ruminococcus2* (OR = 1.846, 95% CI: 1.261–2.704, *P* = 0.002), *Genus Subdoligranulum* (OR = 1.907, 95% CI: 1.165–3.121, *P* = 0.010), *Phylum Verrucomicrobia* (OR = 1.309, 95% CI: 1.027–1.668, *P* = 0.029) on TC risk, while *Class Betaproteobacteria* (OR = 0.522, 95% CI: 0.310–0.879, *P* = 0.015), *Family Family XI* (OR = 0.753, 95% CI: 0.577–0.983, *P* = 0.037), *Genus Sutterella* (OR = 0.596, 95% CI: 0.381–0.933, *P* = 0.024) might be correlated with a decreased risk of TC. Subsequently, various sensitivity analyses indicated no heterogeneity, directional pleiotropy or outliers. In addition, reverse analysis demonstrated a negative causal effect of TC risk on the abundance of the gut microbiota (*Genus Ruminococcus2*, OR = 0.947, 95% CI: 0.907–0.989, *P* = 0.014).

**Conclusion:**

Genetic evidence suggested that bidirectional causal associations of specific bacteria taxa and the risk of TC, highlighting the association of the “gut-thyroid” axis. Further exploration of the potential microbiota-related mechanisms might have profound implications for public health in terms of the early prevention and treatment of TC.

## 1 Introduction

Thyroid cancer (TC) is the most common malignant tumour of the human endocrine system and the head and neck (1). According to World Health Organization, there were an estimated 567,000 new cases and 41,000 deaths from TC worldwide in 2018 (2). The incidence of TC ranked ninth among all tumors worldwide, and the prevalence was more significant in women and people aged 50 years (3). In the United States, the overall incidence of TC has been observed to increase by 3% annually from 1975 to 2013 (4). Among the various types of TC, papillary thyroid cancer (PTC) is the most common and least aggressive histologic type, contributing to the majority of new cases (5). Over the past 30 years, the incidence of TC in the US has nearly tripled, which has been partly driven by an increase in surveillance and diagnostic testing (6). In 2019, the annual cost of TC was estimated at $1.8 billion to $2.1 billion in the United States, placing a significant clinical and economic burden on society (7). Therefore, the prevention and management of TC has been globally recognized as a crucial public health issue.

The intestinal microbiota is increasingly acknowledged as a pivotal “endocrine organ” and “metabolic regulator” within the human body (8). The microbes, genes, and gene products (proteins, enzymes) of it enable its active involvement in the regulation of metabolism, immunity, and endocrine systems (9). In recent years, there has been a growing body of research on the intestinal microbiota, which has revealed its potential influence on the secretion of the thyroid-stimulating hormone via the hypothalamus-pituitary axis, thereby playing a role in thyroid diseases (10). Additionally, a systematic review found that the gut microbiota can contribute to the accumulation of metabolites which, through specific mechanisms, induce genetic instability in the thyroid, ultimately resulting in tumorigenesis and progression (11). In the quest to elucidate these connections, Zhang et al. conducted a study comparing the gut microbiome changes in individuals with TC, thyroid nodules (TN), and healthy controls. They observed that the relative abundances of *Neisseria* and *Streptococcus* were significantly elevated in both the TC and TN groups compared to the healthy controls, while the abundances of *Butyricimonas* and *Lactobacillus* were found to be decreased (12). Similarly, Feng et al. demonstrated that TC patients exhibited a notable enrichment in 19 genera, including *Shigella*, *Clostridium*, and *Klebsiella*, and reductions in 8 genera such as, *Bacteroides*, *Prevotella*, and *Ruminococcus* when compared to the microbial community composition of healthy controls (13). Nevertheless, it was worth noting that these studies primarily relied on observational and cross-sectional analyses, leaving the question of a causal relationship between gut microbiota composition and TC risk without a consensus.

Mendelian randomization (MR) is an analytical approach that has been widely used in epidemiology to explore causal relationships between an exposure (such as a risk factor or intervention) and an outcome (such as a disease or health outcome) by leveraging genetic variants as instrumental variables (14). Recently, MR analysis has become increasingly popular in epidemiological research due to it offering a way to provide evidence for causality without conducting actual experiments on human subjects (15). The basic principle of MR method relies on the fact that genetic variants are randomly allocated during conception and are generally not influenced by confounding factors or reverse causality problems of observational epidemiological investigations (16). Unlike standard MR, the two-sample bidirectional MR involves the use of summary statistics from two independent datasets to evaluate causal relationships between exposures and outcomes, which enhances statistical power. Moreover, the bidirectional MR investigates causal relationships in both directions between two traits, providing a more comprehensive understanding of the complex interplay between variables. As far as we know, no MR analysis has been published on the causality between gut microbiota and TC risk. Therefore, we aimed to conduct a bidirectional two-sample MR analysis to explore the potential causal relationship between the gut microbiota and the risk of TC. This analysis utilized data from two independent genome-wide association study (GWAS), one for TC from the FinnGen consortium and the other for the gut microbiota composition from the MiBioGen consortium.

## 2 Materials and methods

### 2.1 Study design

**Figure 1:**
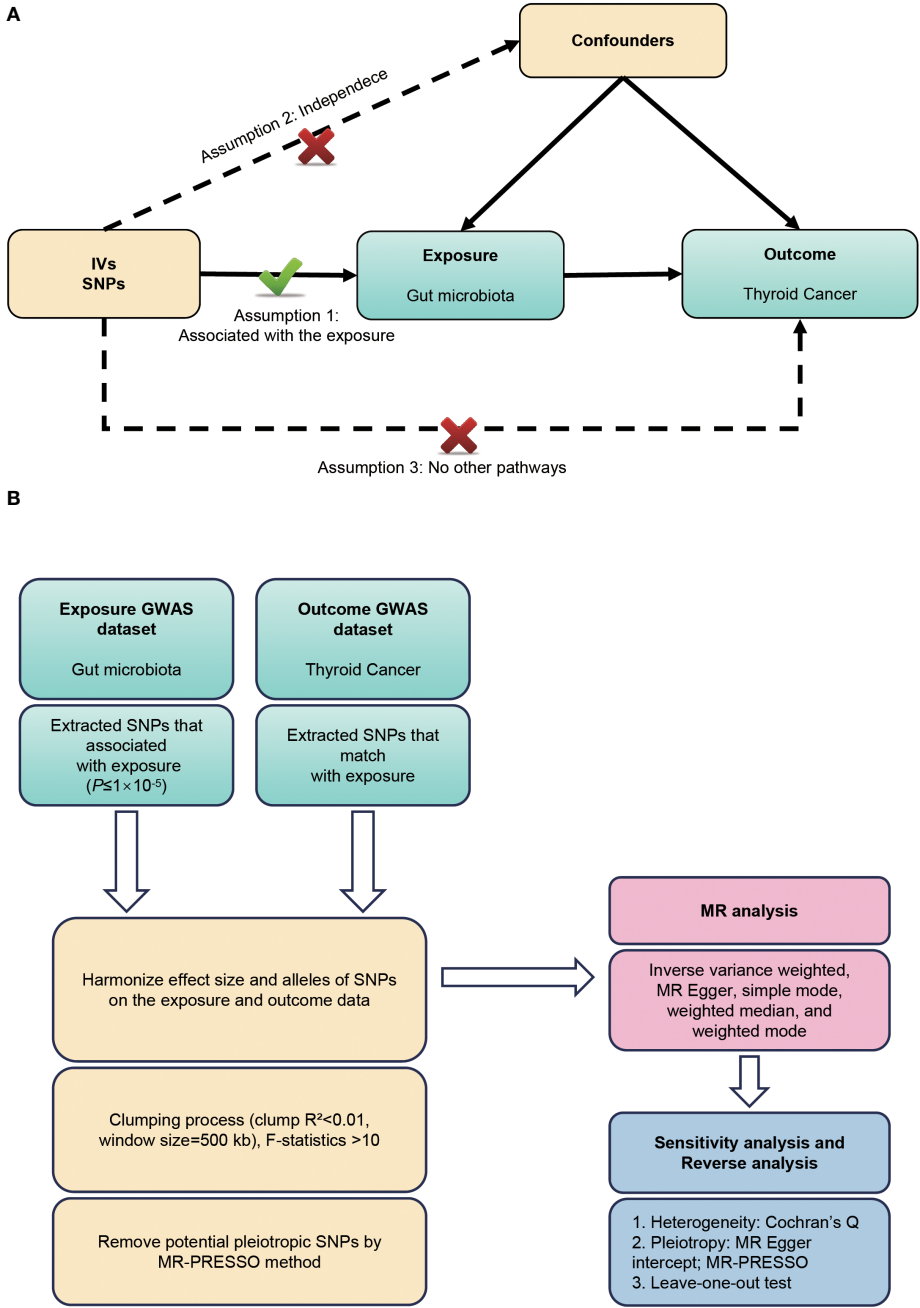
**(A)** Three assumptions of Mendelian randomization. **(B)** Flowchart of this Mendelian randomization study. MR, Mendelian randomization; SNP, single nucleotide polymorphism; GWAS, genome-wide association studies.

This study employed a bidirectional two-sample MR design to elucidate the potential bidirectional causal relationship between gut microbiota and TC risk. The investigation involves two main stages: in the first stage, we employed single nucleotide polymorphisms (SNPs) associated with gut microbiota as instrumental variables to estimate the causal effect of gut microbiota on the TC risk. In the second stage, we used SNPs associated with TC to examine changes in gut microbiota following the development of TC. This dual approach allowed us to explore and infer the crosstalk between the gut microbiota and TC risk in a comprehensive manner. To ensure the validity of the MR analysis, this study follows the three key hypotheses outlined by Bownden et al. (17): (1) The instruments of genetic variations should be strongly correlated with exposure (gut microbiota); (b) The genetic variations should not be linked with any confounding factors related to both gut microbiota and TC; (c) The genetic variations should affect TC solely through gut microbiota, not via other pathways (**Figure 1A**). To uphold ethical standards, all studies included in the GWASs referenced in the analysis were approved by relevant review committees. The flowchart of our work is presented in **Figure 1B**.

### 2.2 Sources of genome-wide association studies

The data on the exposure and outcome in the bi-directional MR study came from the GWAS database. The GWAS summary data of gut microbiota was extracted from a large-scale association analyses that included genome-wide genotypes and 16S fecal microbiome data from 18,340 European individuals (MiBioGen Consortium). Based on the variation of gut microbiota in different populations, the GWAS study eventually yielded 122,110 variant sites from 211 taxa (from genus to phylum level) (18).

The FinnGen research project is a large-scale, population-based initiative in Finland that aims to enhance our understanding of the genetic basis of various diseases and health-related traits. The project involves collecting and analyzing genomic data from a diverse population of Finnish individuals in order to identify genetic variants associated with various health conditions. In our study, we selected the summary data associated with malignant neoplasm of thyroid gland (all cancers excluded) from a FinnGen consortium GWAS containing a sample size of 1,525 cases and 259,583 controls. Importantly, the data employed for this study emanated from the most recent release of the FinnGen consortium (dated December 2022). This latest dataset has been instrumental in our pursuit of examining the connection between gut microbiota and TC, enhancing the accuracy and relevance of our findings (https://gwas.mrcieu.ac.uk/datasets/finn-b-C3_THYROID_GLAND_EXALLC/). Detailed characteristics of source datasets in the study were shown in **Table 1**.

**Table 1:** Detailed characteristics of source datasets in the study.

Trait	Consortium	Population	Sex	Number of SNPs	Sample size
Exposure					
Gut microbiota	MiBioGen Consortium (PMID: 33462485)	European	Males and Females	5,708,796	14,306
Outcome					
Thyroid cancer	FINNGEN	European	Males and Females	16,380,316	1,525

GWAS, genome-wide association studies; SNP, single-nucleotide polymorphism; PMID, PubMed unique identifier.

### 2.3 Instrumental variable selection

All the SNPs selected as instrumental variables in the MR study should obey the three basic assumptions (19). (1) Firstly, we selected instrumental variables that were closely related to the exposure factors (the inclusion criteria of *P* value < 5×10^-8^) for the MR analysis to satisfy the relevance assumption. To include sufficient instrumental variables for screening, we used a more lenient threshold (*P* value < 1×10^-5^) in studies. (2) Then, we set the linkage disequilibrium (LD) threshold of r^2^ < 0.001 and LD distance > 10,000 kb to avoid the offset caused by LD between the variables of interest in the results. (3) To prevent potential pleiotropy, PhenoScanner V2 databases were also used to further verify whether the instrumental variables mentioned above were related to other confounding factors. (4) Finally, we eliminated instrumental variables with an F-statistic < 10 to minimize potential weak instrument bias.

### 2.4 Statistical analysis

In this MR analysis, the inverse variance weighted (IVW) method was used as the primary approach to reveal the potential causation between gut microbiota and TC since it was deemed the most reliable method if there was no indication of pleiotropy (20). The IVW method had a strong ability to detect causality based on the fundamental premise that all genetic variants are valid instrumental variables. In addition, the weighted median, MR Egger, simple mode, and weighted mode methods were used as additional methods to estimate causal effects under different conditions. The MR-Egger method allows researchers to assess whether the causal effect estimate is biased due to pleiotropy and provides a “pleiotropy-corrected” causal estimate when pleiotropy is present. The weighted median method provides a robust estimate of causal effects by calculating the median of causal estimates from genetic instruments while considering their precision (21).

The study utilized the MR-Egger regression to assess the impact of gene pleiotropy on bias, while the MR-PRESSO method was applied to identify and correct for potential outliers resulting from horizontal polymorphisms (22). The MR-Egger intercept test was utilized to evaluate the presence of horizontal pleiotropy (23). In addition, we employed Cochran’s Q test to identify heterogeneity in the included instrumental variables (24). Furthermore, we employed the leave-one-out method to examine the influence of individual SNPs on the causal association determined through MR analysis.

Ultimately, reverse-direction MR was also conducted to identify reverse causality. all MR analyses were performed with the R package of “TwoSampleMR” and “MR-PRESSO” in the R statistical software (version 4.1.3).

## 3 Results

### 3.1 Selection of instrumental variables

First of all, 14,587 SNPs correlated with the gut microbiota were identified as IVs from the MiBioGen Consortium at a relatively loose significance level (*P* < 1×10^-5^) (**Supplementary Table 1**). It contained 211 bacterial traits, including 131 genera, 35 families, 20 orders, 16 classes, and 9 phyla. After a series of quality control steps, 2,236 SNPs were finally included in the analysis. In addition, the F-value of each instrumental variable is greater than 10.

### 3.2 Causal effect of gut microbiota on the risk of thyroid cancer (locus-wide significance level, *P* < 1×10^-5^)

According to the IVW analysis, genetically predicted *Family Christensenellaceae* (OR = 1.664, 95% CI: 1.103–2.511, *P* = 0.015), *Family Victivallaceae* (OR = 1.268, 95% CI: 1.009–1.594, *P* = 0.042), *Genus Methanobrevibacter* (OR = 1.505, 95% CI: 1.049–2.159, *P* = 0.027), *Genus Ruminococcus2* (OR = 1.846, 95% CI: 1.261–2.704, *P* = 0.002), *Genus Subdoligranulum* (OR = 1.907, 95% CI: 1.165–3.121, *P* = 0.010), *Phylum Verrucomicrobia* (OR = 1.309, 95% CI: 1.027–1.668, *P* = 0.029) were positively associated with TC risk. In contrast, genetically predicted abundance of *Class Betaproteobacteria* (OR = 0.522, 95% CI: 0.310–0.879, *P* = 0.015), *Family Family XI* (OR = 0.753, 95% CI: 0.577–0.983, *P* = 0.037), *Genus Sutterella* (OR = 0.596, 95% CI: 0.381–0.933, *P* = 0.024) was inversely correlated to TC risk (**Table 2**). Similarly, the MR estimates of the weighted median indicated that elevated levels of *Family Christensenellaceae* (OR = 1.893, 95% CI: 1.068–3.354, *P* = 0.029) and *Family Family XI* (OR = 0.686, 95% CI: 0.482–0.977, *P* = 0.036) were associated with elevated and reduced risk risk of TC, respectively (**Table 2**). The MR results of all gut microbiota on TC were presented in detail in **Supplementary Table 2**.

**Table 2:** MR results of causal effects between gut microbiome and the risk of thyroid cancer.

**Group**	**Bacterial traits**	**Nsnp**	**Methods**	**SE**	**OR (95% CI)**	***P-*value**
*Class*	*Betaproteobacteria*	11	MR Egger	0.888	0.850 (0.149, 4.844)	0.859
			Weighted median	0.344	0.561 (0.286, 1.101)	0.093
			Inverse variance weighted	0.266	0.522 (0.310, 0.879)	0.015
			Simple mode	0.550	0.587 (0.200, 1.726)	0.356
			Weighted mode	0.520	0.641 (0.231, 1.778)	0.413
*Family*	*Christensenellaceae*	11	MR Egger	0.407	1.840 (0.829, 4.084)	0.168
			Weighted median	0.292	1.893 (1.068, 3.354)	0.029
			Inverse variance weighted	0.210	1.664 (1.103, 2.511)	0.015
			Simple mode	0.465	2.131 (0.857, 5.302)	0.135
			Weighted mode	0.355	1.984 (0.990, 3.977)	0.082
*Family*	*Family XI*	8	MR Egger	0.872	0.366 (0.066, 2.024)	0.293
			Weighted median	0.180	0.686 (0.482, 0.977)	0.036
			Inverse variance weighted	0.136	0.753 (0.577, 0.983)	0.037
			Simple mode	0.267	0.658 (0.390, 1.111)	0.161
			Weighted mode	0.267	0.658 (0.390, 1.110)	0.161
*Family*	*Victivallaceae*	13	MR Egger	0.573	1.441 (0.469, 4.432)	0.537
			Weighted median	0.153	1.272 (0.942, 1.718)	0.117
			Inverse variance weighted	0.117	1.268 (1.009, 1.594)	0.042
			Simple mode	0.255	1.309 (0.794, 2.158)	0.312
			Weighted mode	0.224	1.323 (0.853, 2.053)	0.235
*Genus*	*Methanobrevibacter*	6	MR Egger	0.690	2.140 (0.554, 8.272)	0.332
			Weighted median	0.233	1.536 (0.973, 2.424)	0.065
			Inverse variance weighted	0.184	1.505 (1.049, 2.159)	0.027
			Simple mode	0.305	1.559 (0.858, 2.832)	0.205
			Weighted mode	0.287	1.559 (0.888, 2.736)	0.183
*Genus*	*Ruminococcus2*	15	MR Egger	0.469	1.894 (0.755, 4.752)	0.197
			Weighted median	0.270	1.638 (0.965, 2.780)	0.067
			Inverse variance weighted	0.195	1.846 (1.261, 2.704)	0.002
			Simple mode	0.398	1.515 (0.694, 3.304)	0.315
			Weighted mode	0.341	1.661 (0.851, 3.242)	0.159
*Genus*	*Subdoligranulum*	11	MR Egger	0.648	1.263 (0.355, 4.494)	0.727
			Weighted median	0.349	1.746 (0.882, 3.457)	0.110
			Inverse variance weighted	0.251	1.907 (1.165, 3.121)	0.010
			Simple mode	0.611	1.518 (0.458, 5.027)	0.510
			Weighted mode	0.561	1.533 (0.510, 4.602)	0.464
*Genus*	*Sutterella*	12	MR Egger	0.989	0.658 (0.095, 4.573)	0.681
			Weighted median	0.300	0.622 (0.345, 1.118)	0.113
			Inverse variance weighted	0.229	0.596 (0.381, 0.933)	0.024
			Simple mode	0.509	0.794 (0.293, 2.154)	0.659
			Weighted mode	0.514	0.725 (0.265, 1.986)	0.545
*Phylum*	*Verrucomicrobia*	12	MR Egger	0.546	2.241 (0.768, 6.540)	0.170
			Weighted median	0.161	1.321 (0.963, 1.812)	0.084
			Inverse variance weighted	0.124	1.309 (1.027, 1.668)	0.029
			Simple mode	0.283	1.463 (0.840, 2.549)	0.206
			Weighted mode	0.249	1.463 (0.899 2.382)	0.154

SNP, single nucleotide polymorphism; MR, mendelian randomization; SE, standard error; 95% CI, 95% confidence interval.

### 3.3 Sensitivity analysis

**Figure 2:**
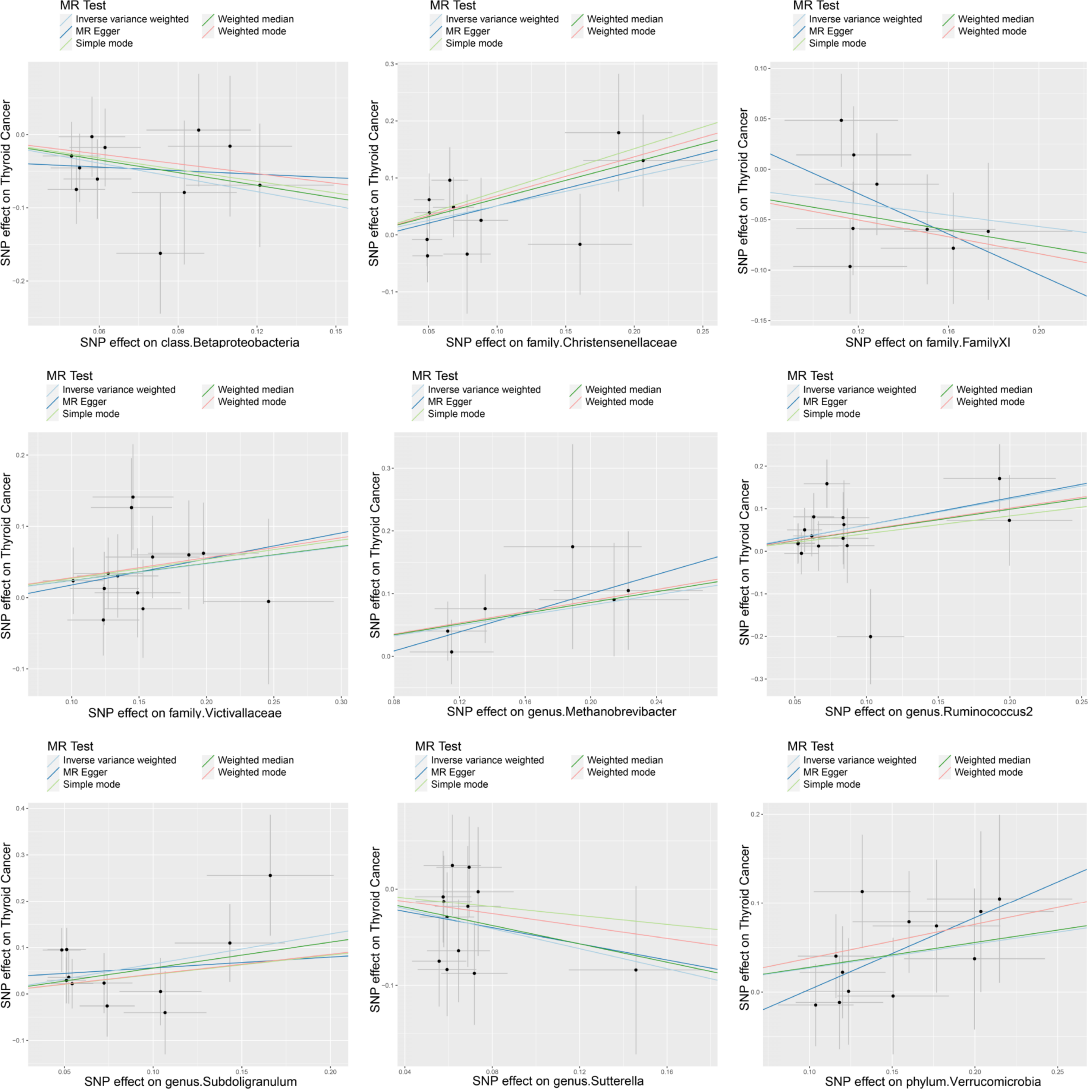
Scatter plots of significant causality of exposure (Specific gut microbiome) and outcome (thyroid cancer risk). The dots represent the effect size of each SNP on each bacterial taxon (x-axis) and thyroid cancer (y-axis), and the grey crosses represent the standard errors. Regression slopes show the estimated causal effect of each bacterial taxon on thyroid cancer. The light blue, dark blue, light green, dark green, and red regression lines represent the inverse variance weighted method, MR-Egger regression, simple mode, weighted median method, and weighted mode, respectively.

**Figure 3:**
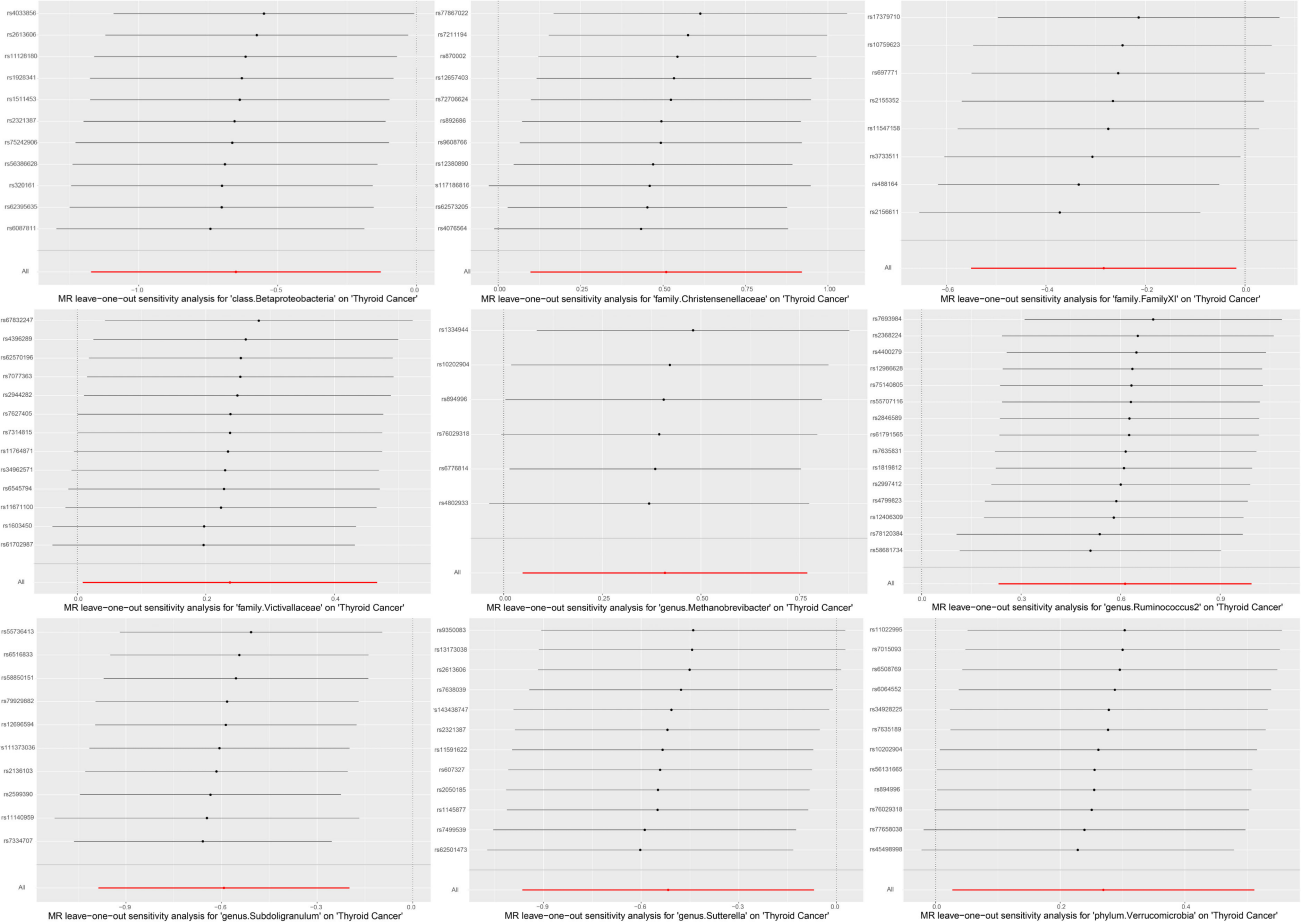
Leave-one-out stability tests causal estimates of exposure (Specific gut microbiota) on outcome (thyroid cancer risk). Circles indicate MR estimates for gut microbiota on thyroid cancer using inverse-variance weighted fixed-effect method if each SNP was omitted in turn.

A series of sensitivity analyses were conducted to assess the heterogeneity and horizontal pleiotropy of the selected instrumental variables. Based on Cochran’s Q test, we observed no significant heterogeneity (*P* > 0.05) (**Table 3**). All *P* values of the MR-Egger intercept tests were > 0.05, indicating no horizontal pleiotropy. Additionally, no outliers were identified through the MR-PRESSO global test (**Table 3**). Detailed scatter plots for each MR method analysis were shown in **Figure 2**. And results from a leave-one-out test suggested that no SNP exerted influential outlier effects (**Figure 3**).

**Table 3:** Sensitivity analysis of the MR analysis results of gut microbiota and the risk of thyroid cancer.

Outcome	Bacterial traits	Cochran Q statistic	Heterogeneity *P*-value	MR-Egger Intercept	Intercept *P*-value	MR-PRESSO Global test *P*-value
Thyroid cancer	*Class Betaproteobacteria*	4.519	0.921	-0.035	0.579	0.935
	*Family Christensenellaceae*	6.758	0.748	-0.010	0.780	0.760
	*Family FamilyXI*	6.803	0.450	0.006	0.435	0.492
	*Family Victivallaceae*	6.716	0.876	-0.019	0.824	0.886
	*Genus Methanobrevibacter*	1.145	0.950	-0.052	0.624	0.736
	*Genus Ruminococcus2*	12.515	0.565	-0.002	0.954	0.619
	*Genus Subdoligranulum*	8.584	0.572	0.033	0.508	0.599
	*Genus Sutterella*	6.543	0.835	-0.007	0.920	0.840
	*Phylum Verrucomicrobia*	4.817	0.940	-0.078	0.336	0.951

MR-Egger, Mendelian randomization Egger.

### 3.4 Causal effect of gut microbiota on the risk of thyroid cancer (genome-wide statistical significance level, *P* < 5×10^-8^)

A total of 1,394 SNPs were identified as instrumental variables at genome-wide statistical significance level (*P* < 5×10^-8^) (**Supplementary Table 3**). When MR analysis was conducted with gut microbiome as a whole, IVW results indicated no evidence of a causal association between the gut microbiota and TC risk (**Table 4**). Additionally, Cochran’s Q test results revealed no significant heterogeneity (*P* = 0.932) and the MR-Egger intercept shows no evidence of horizontal pleiotropy between total gut microbiome and TC risk (*P* = 0.973). Finally, we also did not discover any outliers through the MR-PRESSO global test (**Table 4**).

**Table 4:** MR estimates for the association between total gut microbiota and the risk of thyroid cancer.

Bacterial traits (exposure)	Traits (outcome)	Nsnp	Methods	SE	β (95% CI)	*P-*value	Cochran Q statistic	Heterogeneity *P*-value	MR-Egger Intercept	Intercept *P*-value	MR-PRESSO Global test *P*-value
*Total*	Thyroid cancer	12	MR Egger	0.506	0.970 (0.360, 2.614)	0.953	4.983	0.932	0.002	0.973	0.939
			Weighted median	0.183	1.019 (0.712, 1.459)	0.916					
			Inverse variance weighted	0.143	0.986 (0.744, 1.306)	0.923					
			Simple mode	0.278	1.060 (0.614, 1.829)	0.839					
			Weighted mode	0.226	1.020 (0.655, 1.589)	0.930					

SNP, single nucleotide polymorphism; MR, mendelian randomization; SE, standard error; 95% CI, 95% confidence interval.

### 3.5 Reverse analysis of causality between thyroid cancer risk and gut microbiome

In our study, we utilized reverse causality to explore the relationship between TC incidence and gut microbiota. According to the IVW method, TC risk had a negative causal effect on the gut microbiota (*Genus Ruminococcus2*) in our study (OR = 0.947, 95% CI: 0.907–0.989, *P* = 0.014). However, the remaining 8 gut microbiota indicated no significant causal association with TC risk, including *Class Betaproteobacteria* (*P* = 0.978), *Family FamilyXI* (*P* = 0.337), *Family Victivallaceae* (*P* = 0.819), *Genus Methanobrevibacter* (*P* = 0.309), *Genus Subdoligranulum* (*P* = 0.938), *Genus Sutterella* (*P* = 0.656), *Phylum Verrucomicrobia* (*P* = 0.533) (**Table 5**).

**Table 5:** Reverse analysis of causal effects between thyroid cancer and gut microbiome.

Exposure	Outcome	Nsnp	Methods	SE	OR (95% CI)	*P-*value
Thyroid cancer	*Class Betaproteobacteria*	2	Inverse variance weighted	0.020	1.001 (0.960, 1.042)	0.978
Thyroid cancer	*Family FamilyXI*	1	Inverse variance weighted	0.056	1.055 (0.945, 1.178)	0.337
Thyroid cancer	*Family Victivallaceae*	2	Inverse variance weighted	0.043	0.990 (0.909, 1.079)	0.819
Thyroid cancer	*Genus Methanobrevibacter*	2	Inverse variance weighted	0.046	0.955 (0.873, 1.044)	0.309
Thyroid cancer	*Genus Ruminococcus2*	2	Inverse variance weighted	0.022	0.947 (0.907, 0.989)	0.014
Thyroid cancer	*Genus Subdoligranulum*	2	Inverse variance weighted	0.021	1.002 (0.962, 1.043)	0.938
Thyroid cancer	*Genus Sutterella*	2	Inverse variance weighted	0.026	1.012 (0.961, 1.065)	0.656
Thyroid cancer	*Phylum Verrucomicrobia*	2	Inverse variance weighted	0.034	71.021 (0.956, 1.091)	0.533

SNP, single nucleotide polymorphism; MR, mendelian randomization; SE, standard error; 95% CI, 95% confidence interval.

## 4 Discussion

To our knowledge, we determined the causal influence of gut microbiota on the risk of TC by MR analysis for the first time. In our bidirectional MR analysis, we identified several gut microbiota that exhibit causal relationships with TC risk. Specifically, *Family Christensenellaceae*, *Family Victivallaceae*, *Genus Methanobrevibacter*, *Genus Ruminococcus2*, *Genus Subdoligranulum*, and *Phylum Verrucomicrobia* were correlated with an elevated risk of TC. And *Class Betaproteobacteria*, *Family Family XI*, *Genus Sutterella* exhibited a potential protective effect against TC. Moreover, our reverse analysis indicated that an increased risk of TC could potentially lead to a lower abundance of the *Genus Ruminococcus2.* This study opened up exciting new avenues for further research in the field of gut microbiota and its potential impact on TC. By shedding light on the role of specific gut microbiota in TC risk, our findings might pave the way for future investigations into targeted interventions aimed at modulating the gut microbiota to prevent or manage TC.

The gut microbiota is a large group of microbes found in the human gastrointestinal tract, and plays a pivotal role in maintaining intestinal homeostasis (25). Disruptions in gut microbiota balance can lead to intestinal epithelial barrier dysfunction, resulting in inflammation, hormone inactivation, and even tumorigenesis (26). Recently, the relationship between thyroid function and the gut microbiota has emerged as a prominent area of research. Several studies have indicated that the gut microbiota can influence the secretion of the thyroid-stimulating hormone through the hypothalamus-pituitary axis, thereby potentially impacting thyroid diseases development (10). On the one hand, modifications in the composition, structure, and metabolites of gut microbiota can directly or indirectly affect the activation and cytokine production of immune cells, potentially influencing lymphopoiesis and exerting cancer-promoting effects in TC (27). On the other hand, gut microbiota may influence the metabolism and bioavailability of certain nutrients, affecting iodine absorption and utilization by the thyroid gland. As iodine is an essential component of thyroid hormone synthesis, any interference with its availability may have implications for thyroid function and potentially even contribute to the development of TC (28). Thus, further research in this area is essential to unravel the precise mechanisms underlying the gut-thyroid axis and its significance in thyroid health and disease prevention.

In our study, we conducted MR and sensitivity analysis on the filtered qualifying instrumental variables, and we identified a causal relationship between nine gut microbiota and TC risk. According to a multi-omics study, the abundance of *Christensenellaceae* and *Eubacterium*, both of which are closely related to lipid metabolism, were significantly reduced in the TC group compared to healthy controls (29). In addition, Ishaq et al. demonstrated that compared to healthy groups, the abundance of *Subdoligranulum*, *Verrucomicrobia*, and *Ruminococcus2* were significantly increased, while the abundance of *Prevotella9*, *Bacteroides*, and *Klebsiella* were significantly decreased in the TC group, further accentuating the microbial differences (30). *Betaproteobacteria*, one of the five Classes within the *Proteobacteria* (31). Within a harmoniously balanced gut microbiota that characterized by robust stability, the interaction with the host’s immune system often curtails unregulated proliferation of *Proteobacteria*. However, as observed in this study, the surge of *Proteobacteria* indicates an unstable gut microbial community, which is associated with certain disease states (32). Similarly, a cross-sectional study found that elevated levels of *Proteobacteria* in the majority of TCs by fractional analysis, which may indicate that dysregulation of intestinal ecology is associated with the underlying pathogenesis of TC (33). In addition, we have also found a causal relationship between *Family Victivallaceae*, *Genus Methanobrevibacter*, and *Family Family XI* with the risk of TC. However, there was limited research on the role of these three groups of bacteria in TC. Thus, it was necessary to further study the possible role of *Family Victivallaceae*, *Genus Methanobrevibacter*, and *Family Family XI* in TC development.

Interestingly, our reverse MR analysis revealed that TC may induce a decrease in the of *Genus Ruminococcus2*. Among the dominant genera in our studied population, *Ruminococcus2* holds a noteworthy place as both a symbiotic bioindicator of human health and a producer of short-chain fatty acids (SCFAs) (34). SCFA-producing genera are typically found in abundance within the human gut, benefiting the host by participating in the fermentation of carbohydrates into SCFAs, including acetate and propionate (35). It has been reported that the diminishment of SCFA-producing bacteria may promote the development of TC (33). In light of this, it was plausible to hypothesize that a reduced abundance of *Ruminococcus2* could potentially disrupt the balance of the gut microbial ecosystem, either preceding or following the development of TC. Additionally, more and more researches suggested that the abundance of *Ruminococcus2* tends to be lower in individuals with TC, which indicates *Ruminococcus2* maybe a potential regulator. Therefore, we hypothesized that there is a dynamic interaction between the incidence of TC and the level of *Ruminococcus2*.

Our research had various strengths. First of all, we employed MR analysis to deduce that the relationship between the gut microbiome and the risk of TC is less vulnerable to confounding and reverse causation in comparison to traditional observational analyses. Furthermore, we examined the causal impact of each taxon on TC risk, ranging from the genus to the phylum level, which provides guidance for the prevention and treatment of TC by targeting distinct gut microbiota during clinical practice. Nonetheless, our study also possessed certain limitations that should be acknowledged. To begin with, the identified TC risk variants only account for a small portion of the disease risk, and the intricate correlation structure among gut microbial taxa is confounded by factors such as diet and the possibility of horizontal gene transfer. Secondly, due to the absence of demographic data in the original study, we were unable to conduct additional subgroup analyses to obtain more specific correlations. Thirdly, while MR studies have been considered as excellent proxies of clinical/experimental trials in uncovering causal relationships. However, MR studies could not completely replace clinical trials, as their randomization process was subject to certain limitations such as pleiotropy, GWAS power, and representativeness of the sample, which will inevitably lead to a degree of confounding into the results. Furthermore, the GWAS database of gut microbiota and thyroid cancer selected in this study were two independent datasets, the diversity of gut microbiota in the study population was not analyzed. In the future, further large-scale prospective studies are necessary to investigate the specific role of gut microbiota richness and diversity in TC. Last but not least, this study exclusively focused on individuals of European descent, necessitating replication in other populations to validate these findings.

## 5 Conclusion

Our study provided evidence of a potential causal relationship between specific gut microbiota and TC risk through the bi-directional MR analysis. These findings provided new insights into the prevention, progression, and treatment of TC through targeting specific gut microbiota. In the future, more clinical trials and mechanism studies are needed to explore the exact mechanisms underlying the interactions between the gut microbiota and the prevalence of TC.

## Data availability statement

The original contributions presented in the study are included in the article/**Supplementary Material**. Further inquiries can be directed to the corresponding author.

## Author contributions

XS: Data curation, Methodology, Writing – original draft, Writing – review & editing. SC: Data curation, Methodology, Writing – original draft, Writing – review & editing. SZ: Data curation, Methodology, Writing – original draft. JW: Data curation, Formal analysis, Investigation, Writing – original draft. HC: Funding acquisition, Project administration, Writing – review & editing.
